# Exploring the role of ketone bodies in the diagnosis and treatment of psychiatric disorders

**DOI:** 10.3389/fpsyt.2023.1142682

**Published:** 2023-04-17

**Authors:** Naomi Elyse Omori, Mantas Kazimieras Malys, Geoffrey Woo, Latt Mansor

**Affiliations:** ^1^Health Via Modern Nutrition Inc. (H.V.M.N.), San Francisco, CA, United States; ^2^Department of Psychological Medicine, Institute of Psychiatry, Psychology and Neuroscience, King’s College, London, United Kingdom

**Keywords:** ketone, BHB, β-hydroxybutyric acid, anxiety, depression, schizophrenia, treatment, diagnosis

## Abstract

In recent times, advances in the field of metabolomics have shed greater light on the role of metabolic disturbances in neuropsychiatric conditions. The following review explores the role of ketone bodies and ketosis in both the diagnosis and treatment of three major psychiatric disorders: major depressive disorder, anxiety disorders, and schizophrenia. Distinction is made between the potential therapeutic effects of the ketogenic diet and exogenous ketone preparations, as exogenous ketones in particular offer a standardized, reproducible manner for inducing ketosis. Compelling associations between symptoms of mental distress and dysregulation in central nervous system ketone metabolism have been demonstrated in preclinical studies with putative neuroprotective effects of ketone bodies being elucidated, including effects on inflammasomes and the promotion of neurogenesis in the central nervous system. Despite emerging pre-clinical data, clinical research on ketone body effectiveness as a treatment option for psychiatric disorders remains lacking. This gap in understanding warrants further investigating, especially considering that safe and acceptable ways of inducing ketosis are readily available.

## 1. Introduction

Mental health disorders are some of the most prevalent and challenging conditions that are also frequently associated with poor quality of life, reduced life expectancy (1, 2), and a high socioeconomic burden. Widely acknowledged to be underdiagnosed, rates of psychiatric conditions are on the rise worldwide. A key challenge in psychiatry is the comparatively limited options available for the treatment of diseases. Moreso than other specialties, there has been a dearth of psychotropic drugs with truly novel mechanisms of action (MoA) receiving clinical approval in recent years (3). Historically, this can be attributed to both the complexity of psychiatric pathophysiology as well as the inherently challenging nature of neurobiological research, which has only truly entered a renaissance in the last two decades thanks to advances in experimental techniques.

The limited scope of pharmacological options is particularly problematic for treatment-resistant patients, for whom long-term therapeutic options become extremely limited. In diseases like major depressive disorder (MDD), where up to 50% of patients are estimated to respond inadequately to existing treatments (4), this insufficient range in treatment options results in a significant cost to healthcare systems and the societies they serve. Additionally, existing pharmacological options are known to feature highly undesirable and sometimes life-threatening side-effect profiles, including extrapyramidal symptoms (EPS) as seen commonly with typical or first-generation antipsychotics (5, 6) and selected antidepressants, serotonin syndrome as seen in selective serotonin reuptake inhibitors (SSRIs) and serotonin–norepinephrine reuptake inhibitors (SNRIs) (7), hyponatremia as seen with SSRIs (8), weight gain as seen commonly with atypical or second-generation antipsychotics (9), neuroleptic malignant syndrome as seen in both typical and atypical antipsychotics (10), and cardiac problems as seen in tricyclic antidepressants (TCA) (11). As such there is an immediate need for deeper insights into the pathophysiology of psychiatric illnesses, which in turn should facilitate the development of alternative therapies with unique MoAs.

In the last two decades, advances in genomics, in particular genome-wide association studies (GWAS) (12), and metabolomics have lent greater weight to observations drawn from epidemiological studies regarding the importance of inflammatory processes in psychiatric disease. An increasingly sophisticated understanding of inflammatory biomarkers associated with major psychiatric disorders (13) and neurological function has facilitated the design of preclinical and clinical studies that more robustly interrogate the underlying neurobiological processes of neuropsychiatric disease. Subsequent to the insights gleaned from GWAS, there is a growing body of evidence suggesting that individuals with certain neuropsychiatric disorders have altered metabolic profiles. The potential of therapeutic dietary interventions has been anecdotally appreciated as early as the 1910s where clinicians employed ketogenic diets to alleviate symptoms of epilepsy (14). Today, a modern understanding of metabolomics has led to mitochondrial dysfunction, augmented ATP production, and altered electron transport chains (15, 16) among others being identified variably in major depressive disorder (MDD), schizophrenia, and bipolar disorder. This is alongside emerging evidence that certain therapeutic effects of pharmacological interventions like ketamine, approved by the FDA in 2019 for the treatment of MDD, appear to be mediated by mitochondrial energy metabolism (17). The overall implication is that metabolic processes might not only play a role in the pathophysiology of psychiatric diseases but could have a greater influence on pharmacological treatments, both synergistic and deleterious, than previously appreciated.

**Figure 1:**
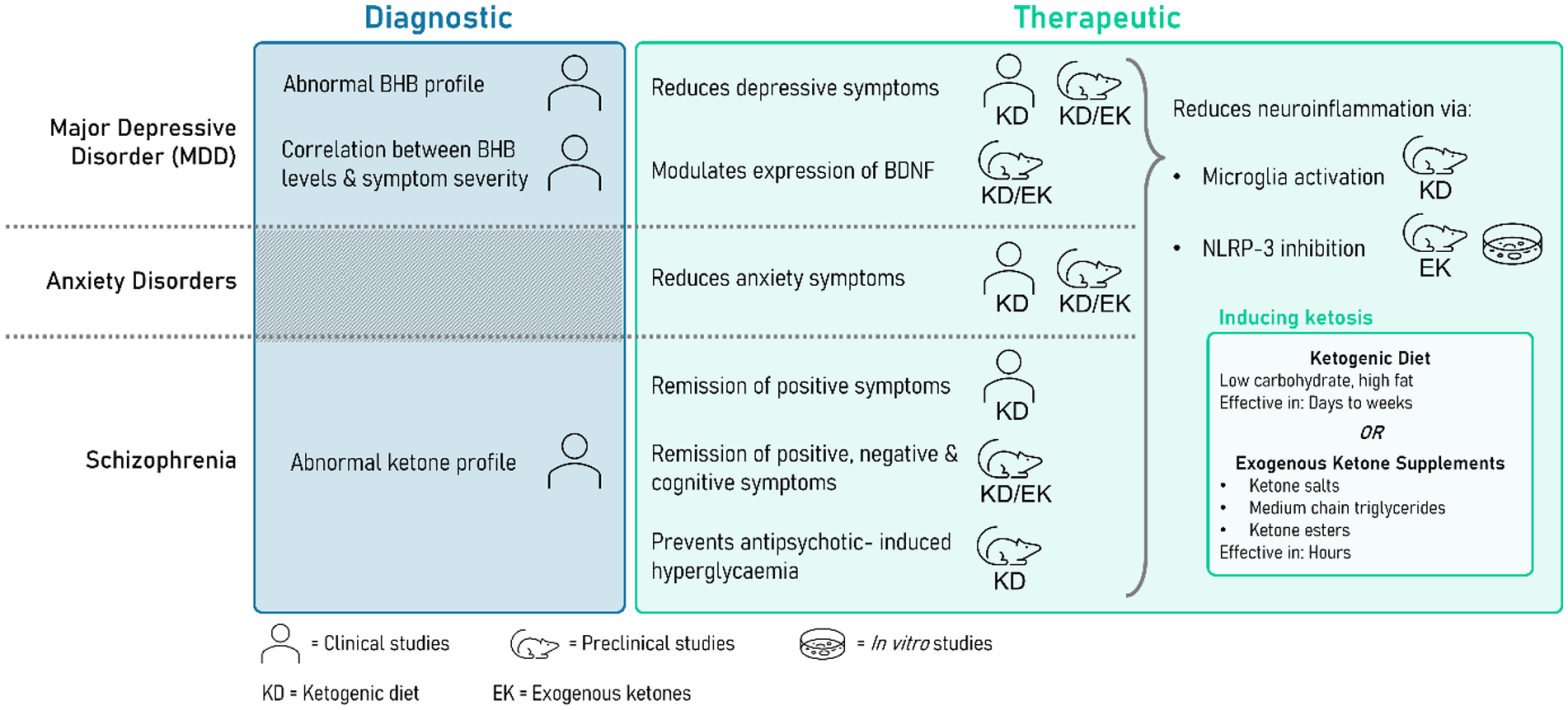
Summarizing the potential diagnostic and therapeutic uses of ketone bodies (KBs). Some clinical studies have shown monitoring levels of KBs may be clinically relevant in establishing psychiatric disease status, severity, and treatment prognosis. A range of preclinical and clinical studies have successfully demonstrated the therapeutic relevance of ketosis as induced by either a ketogenic diet or exogenous supplementation. This summary highlights the limited number of clinical studies focusing on exogenous supplementation.

One metabolic state of particular interest in the treatment and understanding of neurological conditions is ketosis. The neuroprotective effects of ketosis as achieved by exogenous ketone supplementation and a ketogenic diet have been explored in patients suffering from traumatic brain injury (18) and neurodegenerative diseases like Alzheimer’s and Parkinson’s disease (13). The role of ketones in the pathophysiology of mental health conditions is an emerging and highly promising area of psychiatric research. The following review aims to explore recent studies providing insight into the role of ketones in the diagnosis and treatment of three main psychiatric disorders: major depressive disorder (MDD), anxiety, and schizophrenia, which represent some of the most common mental health disorders worldwide. Both the traditional ketogenic diet and more novel exogenous ketone supplementation are considered as part of the review. The subject of much research and an emerging star metabolite of recent literature as a both a proxy biomarker of and potential treatment option for various psychiatric conditions is β-Hydroxybutyrate (BHB), also referred to as 3-Hydroxybutyrate (3HB). Traditionally understood in a metabolic context as an intermediate produced from the breakdown of fat whose elevated presence denotes ketosis, more recent research has elucidated its complex and wide-reaching involvement in many important cellular processes including production of inflammatory cytokines, mediation of oxidative stress, apoptosis, mitochondrial function, ATP synthesis, and glucose metabolism via its involvement with HCAR2, AMPK, and NAD^+^ (19). Particular attention is drawn to its ability to inhibit the NLRP3 inflammasome, which has potentially dramatic implications for its role in inflammation-focused models of psychiatric disease (20) (Figure 1).

## 2. Achieving ketosis

### 2.1. Ketosis and ketone bodies

The section begins with a definitional overview of ketosis. Ketone bodies (KBs) are ketone-containing molecules produced via ketogenesis from the breakdown of fatty acids, the most clinically important of which are acetoacetate (AcAc) and β-Hydroxybutyrate (BHB). Ketosis refers to a systemic metabolic state in which KBs are elevated. Normal human serum circulating levels of KBs are generally less than 0.5 mM. The therapeutic effects of ketosis are most commonly seen within a range of 1–3 mM. Abnormally high levels of KBs, which are generally clinically defined as >3 mM (21), are associated with a pathological metabolic state called ketoacidosis, commonly seen secondary to diabetes, alcohol abuse, and extreme starvation (22). Importantly, ketoacidosis is also accompanied by acidosis (i.e., pH < 7.35), and a raised anion gap (23). Thus, the range for therapeutic ketosis may extend as high as 5 mM in the absence of clinically significant anion gap or pH abnormalities, particularly in neurological treatments (24).

It is possible to achieve ketosis either endogenously or exogenously. Endogenous methods generally refer to dietary augmentation strategies either in the form of a ketogenic diet (KD) or modified feeding patterns. Exogenous methods involve the ingestion of selected formulations of KBs and KB derivates. Generally, adherence to endogenous methods will induce ketosis after a period of days to months (25), while exogenous supplementation can induce ketosis in a matter of hours (26–29).

Two particular ketone bodies (KBs) of interest that play an important role in ketogenesis are β-hydroxybutyrate (βHB) and acetoacetate (AcAc). Ketogenesis is catalysed by 3-hydroxymethylglutaryl-CoA synthase (HMGCS2), a mitochondrial matrix enzyme that generates HMG-CoA from β-oxidation-derived acetoacetyl-CoA and acetyl-CoA. Following the lyase-dependent release of acetyl-CoA, AcAc is formed. AcAc is later reduced to βHB by another mitochondrial matrix enzyme D-βOHB dehydrogenase (BDH1). βHB and AcAc are capable of undergoing interconversion, with βHB oxidizing back to AcAc by BDH1 through mass action. The ratio of circulating βHB and AcAc is regulated largely by the liver and is proportional to the ratio of reduced and oxidized nicotinamide adenine dinucleotide (NADH/NAD^+^; 30). Ketosis is then defined as a metabolic state where the concentration of blood βHB is ≥0.5 mM.

In popular culture dating back to the 1970s, ketosis and KDs have primarily been viewed through the lens of weight loss with its focus on a low carbohydrate, higher protein macronutrient breakdown (31) – an exemplar being the well-advertised “Atkin’s Diet” (32). Subsequent to this, there has been a rise in research focusing on the impact of KDs on obesity, type II diabetes mellitus (T2DM) (33), and the somewhat commercially motivated work on athletic performance enhancement (34, 35). Less widely appreciated by the general public is the KD’s long but under researched history as a therapeutic option for managing neurological conditions like epilepsy (36, 37). More recently, a greater understanding of the metabolic aspects of neurological disease has led to renewed interest in the therapeutic exploitation of ketosis with a focus on exogenous supplementation strategies (38).

### 2.2. Endogenous strategies

#### 2.2.1. KD or “Nutritional ketosis”

The ketogenic diet (KD) is a high fat, low carbohydrate diet that does not provide sufficient carbohydrates to meet metabolic requirements, causing KBs to replace glucose as a preferential energy source. The specific composition of a KD can vary and is best calculated on an individual basis using a 4:1 ratio of fat:protein + carbohydrates as a rough guide (39). The time required to see elevated KB subsequent to the KD varies depending on the model. In rodents, an effect may be seen in as little as 7 days (40) or as much as 3 months (41). In human studies, elevated KBs are rather seen after a period of 3–12 months depending on the subject’s age and previous medical history (42, 43).

A further benefit of the KD is its ability to alter the gut microbiome, which in the context of the evolving understanding of the gut-brain axis in psychiatric research (44–46) could prove particularly meaningful. In terms of overall balance of gut microbiota, there are a series of studies covering a range of age groups that demonstrate the KD’s positive impact on dysbiosis (47–49).

#### 2.2.2. Modified feeding patterns

A similar effect can also be observed via augmenting feeding patterns either by intermittent fasting (IF), caloric restriction (CR), or a combination of the two. Similar to the KD, a rudimentary appreciation of their potential therapeutic value has existed since the early 20^th^ century (50–54). Various protocol exists for intermittent fasting including fasting on alternate days, restricting food intake to a 6–8 h window on a daily basis, and the 5:2 method of fasting on two consecutive days each week (38). Dependent on the protocol, both IF and CR have been shown to increase KBs. In rodent studies, they have also been associated with a range of desirable health outcomes including increased lifespans, weight loss, improved glucose tolerance, and increased lean mass (55–58).

### 2.3. Exogenous ketone supplementation

Exogenous ketone supplementation refers to ingestible ketone preparations that can acutely induce ketosis without the need for long-term dietary modification. They are a controlled and reproducible method for inducing ketosis, making them of particular interest as adherence to KDs can be challenging and in some cases practically unfeasible, which potentially prohibits certain groups from benefitting from the therapeutic or performance enhancing features of ketosis. The three main ketone formulations used in exogenous preparations are ketone salts, medium chain triglycerides (MCTs), and ketone esters and derivatives. Free acid forms of KBs are less stable and are unsuitable for creating preparations that can reliably induce ketosis. From a formulation perspective, each form offers pros and cons, with many commercial preparations utilizing a blend of the three.

#### 2.3.1. Ketone salts

Ketone salts are forms of ketone acids buffered with Na^+^, K^+^, or Ca^2+^. Ketone salts have been shown to modestly elevate KBs when compared to placebo groups to roughly 0.6–1 mM. A benefit is that salts are relatively easily formulated. However, they can cause deleterious electrolyte imbalances with sustained supplementation that can lead to hypernatraemia and gastric hyperosmolarity. This is compounded by the fact that the majority of ketone salts are racemic meaning that only half of the KBs are bioavailable, which results in an effectively doubled salt load.

#### 2.3.2. MCTs

An alternative class of substances are medium chain triglycerides (MCTs), which are KB precursors made up of triglycerides with two to three fatty acids and an aliphatic tail of 6 to 12 carbon atoms. MCTs are a readily available source of high energy. They are often discussed in relation to the closely related structure long-chain triglycerides (LCTs), another widely available type of dietary lipid that is absorbed and metabolized in differently to MCTs. Most notably, MCTs can be metabolized into KBs in a way that LCTs cannot. LCTs are dependent on the fatty acid β-oxidation pathway and are usually hydrolyzed in the gut to form long-chain fatty acids (LCFAs) that re-esterify again into triglycerides and are then transported to the liver via the lymph system. In contrast, MCTs are predominantly absorbed as medium-chain fatty acids (MCFAs) directly into the blood where they undergo first pass hepatic metabolism via the portal vein to form acetyl CoA and KBs. When given as a single energy source (i.e., in the absence of carbohydrates) to healthy human subjects, isotope tracer studies show that around 40% of MCTs are oxidized to CO_2_ which increases to 62% when administered in conjunction with carbohydrates and protein (59). A side-effect of overconsumption of MCTs is gastrointestinal (GI) discomfort (60), which can be addressed by a gradual increase of MCT supplements over an extended period of weeks.

There are indications that combining ketone salts and MCTs could be potentially useful. In a rodent study (61), tested the effects of a 28-day course of an exogenous ketone supplement made up of an Na/K β-hydroxybutyrate (βHB) mineral salt (BMS) in a 1:1 ratio with an MCT oil comprised of approximately 65% caprylic triglyceride and 45% capric triglyceride. When compared with a MCT monotherapy, the combined BMS + MCT supplement was shown to elevate βHB to a similar extent to MCT without causing the GI side-effects observed in the MCT cohort. It is thought that this could be due to the combination of KBs being delivered directly via the salts as well as the sustained ketogenesis being stimulated by the MCT.

#### 2.3.3. Ketone esters and derivatives

Ketone esters are KBs that are bonded by esters to a backbone molecule. These esters have a more stable shelf life and can be broken down by gastric esterases to generate KBs in their free acid form. A commonly utilized backbone molecule in these formulations is 1,3-butanediol, an organic diol previously approved by the Food and Drug Administration (FDA) as a solvent for food flavoring agents. The addition of BHB or AcAc via transesterification can produce ketone esters, which are generally the most energy-dense exogenous ketone supplements gram for gram. While other backbones like glycerol can also be used to formulate ketone esters, the use of 1,3-butanediol is considered superior when formulating EKs for ketosis as it metabolizes completely to BHB, contributing to a sustained ketosis. Some examples of ketone esters and derivatives include the commercialized (*R*)-3-Hydroxybutyl (*R*)-3-hydroxybutyrate ketone monoester (62) and bis-hexanoyl-(R)-1,3-butanediol, and chirally pure R-1,3-butanediol standalone (63–66). Studies have demonstrated that 1,3-butanediol ketone esters can reliably produce dose-dependent ketosis of 1–7 mM with minimal adverse side effects (65).

### 2.4. Metabolic profile of exogenous vs. endogenous ketosis

While both exogenous and endogenous means of achieving ketosis both yield elevated KBs, the fundamental benchmark criteria of ketosis, there are some points of convergence and divergence in the metabolic features of the two methods as previously summarized by Poff et al. (67). Both the KD and exogenous ketones can produce a total KB concentration of approximately 0.5 to 6 mM depending on the composition and duration of the KD and the formulation of the exogenous preparation used. Exogenous ketones can elicit a response within minutes to hours, while the KD typically requires adherence of some days to weeks. The ratio of R-BHB:AcAc is also slightly lower in the KD ranging from 2:1 to 4:1 (21) compared to 3:1 to 6:1 (68) with exogenous supplements.

As a key feature of the KD is reduced carbohydrates, generally less than 5% of the total energy intake, circulating concentrations of glucose are globally lowered during the KD (31), where this is not necessarily the case with exogenous ketone supplementation although there is evidence that they can acutely regulate glycemia by either lowering fasting glucose concentrations or attenuation of postprandial rises in glucose. As this appears to happen independent of insulin concentrations, it is most likely that this regulation occurs as a result of hepatic glucose output rather than an increase in glucose uptake from skeletal muscle.

These points of difference become important in the interpretation of studies discussing the therapeutic and pathophysiological value of KBs and ketosis. It is often the case that mechanistic papers will extrapolate or incorrectly equate the effects of isolated exogenous KB studies to KD-induced ketosis. The move to study molecules separately is understandable from an experimental perspective but can falsely represent the broader metabolic context of ketosis achieved by a KD. A similar caution can be applied to generalized conclusions drawn from *in vitro* studies or conceptual leaps made between clinical and preclinical studies. An example of such seemingly contradictory results are the observed neuroprotective effects imparted by KDs in epilepsy patients (69), which are less reliably replicated in studies that attempt to correlate exogenous KB supplementation with seizure control (70). In this case, it is possible that the increased KB concentration is only one aspect of a multifaceted metabolic profile conferring therapeutic value in epilepsy, for example. Indeed, looking to the future it is likely that the greatest advances in understanding the role of metabolic dysfunction in neuropsychiatric conditions will come with studies that can integrate multiple findings on individual metabolic components to paint a holistic picture that also considers the effects of environmental and genetic factors on systemic cycles.

### 2.5. Cerebral Ketone utilization

While its primary metabolic substrate is certainly glucose, in certain situations the brain has also been found to be capable of fulfilling metabolic requirements by exploiting ketones. Uptake of KBs into the brain appear to be largely dependent on systemic blood concentration (71–73) meaning that ketone utilization increases during periods of low glucose availability (i.e., starvation) as confirmed by PET studies in rats showing that the uptake of AcAc doubles during periods of ketosis (74, 75). Increased cerebral uptake of KBs also occurs when systemic KBs are elevated via exogenous supplementation, with studies of cerebrospinal fluid (CSF) showing that concentrations of βHB increase to become comparable to that of a 24 and 40 h fast after dosing with an exogenous preparation of 4 and 8 g of KBs, respectively, (76). The mechanism by which this happens is not entirely clear, but some rodent studies indicate that KBs enter the brain through monocarboxylate transporters (77). Further, preclinical studies have shown faster recovery in rodents afflicted with TBIs fed a diet of ketone-supplemented glucose (78, 79), implying an important role for ketones in the correct context. The use of ketones theoretically offers a short-term option for the brain to bypass glucose-derived energy metabolism pathways as well as triggering a cascade of protective features that could improve long-term functional outcomes in patients.

## 3. Major depressive disorder

### 3.1. Disease overview

Major depressive disorder (MDD) is a common psychiatric condition characterized by low mood, anhedonia, disrupted appetite and sleep, and impaired cognitive function. Worldwide, it is estimated to affect over 300 million people (80) or around 6% (81) of the population, making it a leading contributor to chronic disease burden. Associated not only with a 60–80% increased risk of mortality (82, 83), longitudinal studies have also identified an elevated risk of cancer, obesity, cardiovascular disease, diabetes, and Alzheimer’s disease, which is indicative of a systemic component affecting multiple organ systems.

The current understanding of MDD, which is indeed the case for many psychiatric disorders, acknowledges the presence of both genetic and epigenetic factors, whose complex interplay makes the pathophysiological understanding of MDD challenging. GWAS indicate a genetic contribution of at least 35% encompassing both hereditary and sporadic rare mutations (84). The lack of consistent findings from GWAS should be noted, indicating that environmental interactions play a significant role in the overall expression of MDD. This is supported by strong correlations for specific environmental factors, particularly during early development, including childhood trauma (85, 86), low educational attainment (87), socioeconomic problems (e.g., unemployment) (88), and recent stressful life events (e.g., bereavement) (89).

Three main biological systems have been explored in studies aiming to understand the pathophysiology of MDD, which are the immune system, the autonomic nervous system, and the hypothalamic–pituitary–adrenal (HPA) axis. Of these, the most widely researched is the HPA axis, although the topic of inflammation is generally a growing area of contemporary interest in neuropsychiatric research. The HPA axis refers to a feedback system of glucocorticoid hormones (i.e., cortisol and corticosterone) that are shuttled between the hypothalamus, pituitary gland, and adrenal glands to dynamically regulate various physiological systems (90), in particular stress reactions, immunity, and fertility. HPA activity is thought to be strongly influenced by environmental factors and disruption in the HPA axis has been observed in a range of physical and mental health conditions. Hyperactivity of the HPA axis with elevated cortisol has emerged as one of the most consistent biological traits of MDD (91–93), although the exact reasons for this remain unclear. The impact of antidepressants and glucocorticoid-lowering agents on HPA hyperactivity are similarly unclear, with studies showing an unclear correlation between their usage and reduction in cortisol (94–97).

An inflammatory model for depression is informed by a growing body of preclinical and clinical evidence demonstrating elevated levels of peripheral inflammatory cytokines, such as TNF and IL-6 (98, 99), which can be transported across the blood–brain barrier (BBB) to directly interact with cerebral units, as well as genes involved in cytokine signaling (100). This is also supported by independent studies finding that patients receiving cytokine treatments like interferon-γ or IL-2 for cancer or hepatitis tend to develop symptoms of MDD (101). Imaging studies have also shown increased neuroinflammation and microglial activation in MDD patients (102, 103). Regarding structural brain alterations, imaging studies have also been able to consistently demonstrate smaller volumes in the basal ganglia, hippocampus, fontal, region, thalamus for MDD, with a note that hippocampal volume reduction has also been observed in bipolar disorder, anxiety disorders, substance-use disorder, and schizophrenia (104).

### 3.2. Role of ketone bodies in pathophysiology of MDD

#### 3.2.1. Abnormal BHB levels in MDD

##### 3.2.1.1. Preclinical

Recently, there has been evidence to suggest that patients with MDD have altered energy metabolism profiles. In particular, BHB has been found to be elevated in studies of depression-associated models. A preclinical study of rats subjected to acute stress found BHB levels to be acutely raised in the prefrontal cortex (PFC) during acute stress responses as measured by the tail-suspension test (TST). The TST is frequently used to screen the potential of antidepressant drugs and involves suspending mice by their tails such that they are unable to escape (105). Over a fixed period, escape-oriented behaviors are quantified with mobility or sustained effortful behavior. A reduction in mobile time is considered a metric of antidepressant success. Acute stress responses are mediated by the HPA axis, hyperactivity of which has been consistently documented in MDD patients. The study found that acute TST-induced stress was accompanied by an increase in serum BHB. Further, exogenous BHB administered to the PFC was associated with an increase in mobile times during TST. While difficult to attribute specifically to MDD pathophysiology, the results appear to show that acute stress responses are accompanied by an increased uptake of KBs and vice versa but only specifically in the PFC. In this case, the authors hypothesized that elevated BHB in the PFC served to stimulate glutamatergic signaling thus modulating stress responses based on previous studies showing that IV BHB rapidly produces glutamate and glutamine in the brain (106), and that the presence of these compounds increased glutamatergic signaling (107).

##### 3.2.1.2. Clinical

In a study that compared adolescent (*n* = 11) and adult patients (*n* = 21) with MDD against healthy age-matched controls, plasma levels of BHB were found to be statistically significantly elevated in both MDD age groups compared to controls with high effect sizes based on power analysis (108). Levels of dityrosine, a protein by-product of oxidative stress, were also found to be elevated in the adult MDD group. MDD patients in this study were noted to be on a range of different medications including fluoxetine, vortioxetine, duloxetine, escitalopram, venlafaxine, vortioxetine, and combinations of the above. Notably, antidepressants did not appear to have a statistically significant effect on the trends observed although the authors acknowledge the small cohort size and short observation periods as factors limiting factors.

Metabolomic studies of plasma metabolites using mass spectrometry provide more comprehensive profiling of MDD patients and are being used more frequently to evaluate the impact of drug therapies (109). In a multicenter study, Setoyama et al. (110) evaluated over 120 blood plasma metabolites from 339 medicated and unmedicated psychiatric patients with a known diagnosis of MDD and found that levels of BHB along with betaine, citrate, creatinine, and gamma-aminobutyric acid (GABA) were positively associated with increased severity of depression irrespective of medication. The implication of this work is that BHB could be used as a biomarker for not only identifying MDD, as proposed by Krivosova et al. (108), but also evaluating severity of depression (SOD). This would offer a more objective method for evaluating SOD, a metric that is otherwise largely assessed via subjective, self-reported questionnaires like the Patient Health Questionnaire (PHQ)-9.

It should be noted that studies not specific to MDD that also report elevated BHB under various test conditions mean that BHB might also be viewed as a broader biomarker of psychosomatic stress rather than MDD specifically. For example, elevated BHB has been reported in preclinical studies (111) as well as clinical studies of normal weight men without a MDD or similar psychiatric diagnosis during psychosocial stress studies (112).

#### 3.2.2. BHB as a predictor of antidepressant therapy success

There are emerging studies that indicate serum BHB levels could be used as a predictor of antidepressant therapy success. Saito et al. (113) recruited 132 patients presenting with psychogenic somatoform symptoms of unknown origin who were not taking any psychotropic medications with the exception of benzodiazepines. Patients were then randomly prescribed the SSRI sertraline or the SNRI venlafaxine with dosage being titrated up to a standard intermediate dose (50 and 75 mg/day respectively) over a fortnight. Serum BHB levels were measured pre- and post-therapy, with post-therapy being defined as the time point at which patients reported subjective improvements of physical symptoms. Baseline serum BHB was found to be high-normal (<80 μmol/L) in 30% of patients. Patients with elevated BHB responded more frequently to treatment and statistically significant decreases in serum BHB were recorded in this group post-therapy. Multiple logistic regression analysis showed that elevated BHB was associated with 44% sensitivity and 94% specificity for effectiveness of treatment with a positive predictive value of 92.5%. Of all symptoms reported, logistic analysis showed that only suicidal ideation was correlated with elevated BHB, which is consistent with earlier studies evaluating the severity of depression and nature of symptoms in psychiatric patients (110). This study also made attempts to correlate serum BHB levels with mood disorders diagnosis or CES-D score but found no statistically significant relationships. The authors instead speculated that elevated BHB might be seen as a generic marker of psychosomatic stress rather than being specific to depression. Interpretation of study results is hampered by the lack of a placebo control group and does not account for reduction in global stress and thus potentially serum BHB as a consequence of receiving ‘care’ in the general sense. Results are also potentially confounded by polypharmacy, which was the case in one third of patients already taking benzodiazepines. Overall, the study highlights an interesting positive predictive potential of BHB for pharmacological treatments.

#### 3.2.3. BHB modulates expression of BDNF

Elevated levels of brain derived neurotrophic factor (BDNF) have been shown to alleviate symptoms of depression and anxiety and yield cognitive improvement. BDNF increase has also been implicated in therapeutic effects of some anti-depressant treatments (114). Hippocampal expression of BDNF has previously been observed as a consequence of exercise in preclinical studies (115–119). There is also long-standing qualitative understanding that exercise appears to impart a positive effect on cognitive performance across a range of age groups (120–123). Building on the understanding that exercise can induce elevated levels of BDNF, Sleiman et al. (124) identified BHB as a BDNF promoter that acts upon HDAC2 and HDAC3. The preclinical work compared BDNF levels from the brains of mice that exercised on a running wheel for 30 days against a control group that did not exercise along with the observation that hippocampal levels of BHB appeared to be higher in the exercising cohort. This hypothesis was further tested by both treating hippocampal slices with BHB in an *ex vivo* study and an *in vivo* study involving intraventricularly injecting exogenous BHB into the non-exercising mice against a saline control, both of which were found to directly induce an increase in BDNF with gene expression analysis specifically identifying an increase in BDNF promoter I activity. This study is important as it offers a mechanistic insight into the role of BHB in cerebral gene expression and highlights the broader effects of peripheral metabolic changes on brain function.

### 3.3. Evidence of ketogenic diet for treatment

#### 3.3.1. Preclinical

An early study in rats found that the during the Porsolt test, subjects that had been subject to a KD had lower immobility times demonstrating a reduction in depressive-behaviors or ‘behavioral despair’ (125). The KD has been found to reduce lipopolysaccharide-induced depressive-like behaviors such as social defeat in preclinical studies of CD-1 mice when subjected to social interaction tests (SIT), tail suspension tests (TST), forced swimming tests (FST), and sucrose preference tests (SPT) (126). KD mice were fed a diet of 10% protein and 90% fat compared to a standard per-calorie diet of 10% protein, 80% carbohydrates, and 10% fat. Along with a reduction in depressive behaviors, a significant reduction in microglial inflammatory activation was observed in the lateral habenula (LHb).

#### 3.3.2. Clinical

In humans, there are limited studies evaluating the efficacy of the KD, although there are case studies that indicate their use can ameliorate not only clinical depression but type II diabetes (T2DM), a common comorbid condition (127). In the study, a 65-year-old woman with a 26-year history of T2DM and MDD demonstrated a rapid resolution of symptoms over a 12-week intervention period of KD with HgA1c reducing to non-diabetic levels and depressive symptoms as assessed by PHQ-9 and GSE/MSC reducing to normal levels.

### 3.4. Evidence of exogenous ketones for treatment

There is a modest but growing body of preclinical research demonstrating exogenously administered BHB’s capacity for attenuating depressive symptoms. It is thought that BHB mediates inhibition of NLR family pyrin domain containing 3 (NLRP3) inflammasomes (20) and confers a neuroprotective effect via inhibition of HDAC function, which in turn modulates expression of BDNF. BHB may also reduce NLRP3-mediated interleukin (IL)-18 and IL-1β production in monocytes. It is also notable that independent studies on the pathophysiology of MDD have identified microglial dysfunction, given that microglia and macrophages have an abundance of HCA2, which is a BHB receptor (128, 129).

Chen et al. (130) evaluated the potential role of BHB in alleviating depressive behaviors. They used two models for inducing MDD including a spatial restraint stress group and a dexamethasone-induced stress group and then compared 12 separate independent test groups receiving either exogenous intraperitoneal BHB injections, a KD, exercise, the TCA imipramine, or saline as interventional measures. Significantly decreased histone H3 (H3k9bhb) was seen in both MDD models and was used as an objective measure of depression. Administration of exogenous BHB was found to improve the behavioral symptoms of depression during tail suspension, forced swimming, and sucrose preference testing and increased levels of H3k9bhb in the hippocampus and hypothalamus. Notably, BHB was found to be increased in the hippocampal region after treatment with both BHB and imipramine, and BHB increased mRNA and protein level expression of BDNF, which is consistent with Sleiman et al.’s (124) findings. KD and exercise groups also showed improvement in depressive behaviors. This study provides greater mechanistic insight by identifying a link between BHB and BDNF expression via H3k9bhb.

Contemporaneously, Yamanashi et al. (131) also reported attenuation in MDD-related stress responses following the exogenous administration of BHB. A chronic unpredictable stress (CUS) model of MDD and immobilization (IMM) stress were used to simulate depression in rats. IL-1β and tumor necrosis factor (TNF)-α were assessed as outcome measures following subcutaneous administration of BHB. In forced swim, sucrose preference, elevated plus maze and novelty suppressed feeding tests, CUS rats showed a statistically significant attenuation of depressive and anxiety behaviors after single dose subcutaneous BHB. Variable results in cytokine readings were obtained across the two rat models. Elevated baseline hippocampal levels of IL-1β in IMM-stressed rats was found to be reduced after BHB injection but were unchanged in the CUS model. TNF-α, while not elevated in IMM-stressed rats was suppressed further after BHB injection and was significantly decreased by BHB in the CUS model. BHB administration did not alter IL-10 expression in either model. The results of Yamanashi et al. (131) are in broad agreement with Chen et al. (130), despite the use of differing rodent models of MDD, and provide an alternative insight into how BHB’s antidepressant effects may be imparted via blockades of NLRP3-generated IL-1β via its cytokine analysis. As NLRP3 was not directly evaluated this remains speculative. An interesting consideration is that Yamanashi et al. (131) demonstrated that peripheral subcutaneous rather than intracerebral injection of BHB was sufficient to induce antidepressant effects.

More recently, Kajitani et al. (132) revisited cytokine analysis during direct injection of BHB into the prefrontal cortex (PFC) of rats subjected to behavioral testing. A CUS-simulated MDD group were compared against a control group during forced swim testing. The CUS group exhibited higher baseline TNF-α in the PFC and BHB was found to decrease this expression. No significant change was seen for IL-1β levels. It should be noted that compared to TNF-α expression, which continues to evolve long after initial response to stress, the temporal response of IL-1β is much more dynamic with compensatory pathways restoring expression to baseline faster than TNF-α. It is possible that the lack of change in IL-1β could be due to experimental designs being unable to capture the likely transitory nature of its attenuation. Overall, the study lends strength to the notion that BHB suppresses neuroinflammation by modulating stress-induced increases in TNF-α and perhaps IL-1β. Through monitoring of corticosterone secretion, they also postulated that CUS modulated the HPA axis response, which was in turn regulated by the injection of BHB. This suggests that BHB might also act on corticosterone receptor expression in the PFC and may play a role in HPA regulation.

The effect of capric acid, an MCT, was also evaluated in a study of mice subjected to behavioral tests, the results of which mimic depressive behaviors (133). An acute high dose (30 mmol/kg) and chronic low dose (0.1–3 mmol/kg) of capric acid solution were administered to mice via oral gavage. Behavior testing including light/dark transition, open field, elevated plus maze (EPM), FST, and TST were performed. A consistent study-wide effect relating to capric acid’s potentially depression-attenuating capacity could not be drawn. The study also found that high dose capric acid killed four subjects and was generally associated with an increase in depressive behaviors in the open field, EPM, and light/dark transition tests, perhaps indicating that the substance is associated with some toxicity in higher doses that overrides any potential effects due to ketosis. The study draws attention to the need for evaluating the tolerability and safety of novel ketone preparations and protocols.

## 4. Anxiety disorders

### 4.1. Disease overview

Anxiety disorders are a group of mental disorders associated with uncontrollable and disproportionate feelings of anxiety, fear, and panic. Common examples of anxiety disorders include: generalized anxiety disorder (GAD), substance-induced anxiety disorder, and to a lesser extent obsessive compulsive disorder (OCD). Anxiety disorders are generally underpinned by a series of physical symptoms (including a rapid or irregular heartbeat, light-headedness, sweating, breathlessness, tremor, feeling hot, and chest pains), mental symptoms (including an inability to relax, feeling nervous, sleep disruptions, intrusive or obsessive thoughts, feeling tearful, and excessive worrying), and behavioral changes (including anhedonia, avoidant behavior, and compulsive behavior). Due to the heterogeneity of anxiety disorders, discussing them together creates some limitations. However, they are discussed together here as a number of preclinical and clinical studies often generically evaluate changes in anxiety-like behaviors or symptoms rather than differentiating based on specific disorders.

Anxiety disorders are some of the most prevalent mental health conditions with some population-based studies estimating that up to a third of individuals will be affected by one in their lifetime (134). Moreso than other psychiatric conditions, anxiety disorders are characterized by common and a diffuse range of symptoms that also exist in healthy, non-afflicted individuals as a normal response to fear-inducing situations, making the distinction between adaptive and non-adaptive fear-responses highly subjective and challenging to draw even for seasoned clinicians. As such, there are some methodological challenges in epidemiological studies assessing prevalence; cross-cultural research has indicated global variability in prevalence but in the case of anxiety disorders it is arguably more difficult to attribute this to cultural factors in studies that have not used standardized diagnostic tools such as the Structured Interview for DSM (SCID), the Diagnostic Interview Schedule (DIS), the Composite International Diagnostic Interview (CIDI) 3.0 for DSM, or the Mini-International Neuropsychiatric Interview (M.I.N.I 6.0). Regardless, there is sufficient evidence to show that anxiety disorders contribute to a large proportion of overall burden of disease.

A pathophysiological understanding of the mechanisms of anxiety disorders remains incomplete. Notably, anxiety disorders are commonly comorbid with MDD, which has led to the equivocal assumption that the two have a shared aetiology; studies on the pathophysiology of anxiety disorders will often discuss depression as related adjunct. Widely thought to play a significant role in various anxiety disorders, dysfunction in the GABA system has been identified as a key element of anxiety disorders particularly following clinical studies of benzodiazepines, barbiturates, and neuroactive steroids, which are all allosteric modulators of the GABA_A_ receptor (135). Abnormalities in serotonin and norepinephrine function are also well established in anxiety disorders, which contribute to the remarkable efficacy of certain SSRIs in the treatment of GAD. Further, region-specific abnormalities in neurotransmitter expression are thought to contribute to specific symptoms with hypersensitive fear or stress responses being attributed to cortical-hippocampal-amygdala pathways, disrupted sleep being associated with thalamic and brainstem dysregulation, and altered appetite and libido being partially governed by hypothalamic dysfunction (136).

Preclinical metabolomic and proteomic studies in mice have found altered energy metabolism associated with divergent mitochondrial function as well as unique biomarkers associated with intermediates of the TCA cycle (137, 138). The regulating effects of the gut microbiota on symptoms of anxiety and depression have also been recognized, in particular the role of microbiota in modulating HPA axis function and direct activation of neuronal stress circuits (139). Recent metabolomic studies have been surprisingly successful in identifying metabolomic alterations unique to anxiety. A Dutch study comparing a cohort of comorbid anxiety-depression against a current depressive or anxiety disorder only cohort identified seven metabolomic alterations unique to depression (specifically inflammatory and atherogenic-lipoprotein), which was found to be similarly deviated in the comorbid group but not altered in the anxiety-only group (140). This indicates whilst substantial clinical overlap is evident, metabolomic profiles are not necessarily shared between the groups. Studies focusing on the role of KBs and the KD in anxiety mainly center around the amelioration of anxiety-like symptoms both preclinically and clinically.

### 4.2. Evidence of the KD for treatment

#### 4.2.1. Preclinical

A preclinical study found that a 6 weeks program of the KD and exercise can lead to a decrease in anxious and depressive behaviors (141). A KD comprised of 81% fat, 16% protein, and 1% carbohydrate compared to a control diet of 13% fat, 25% protein, and 62% carbohydrate was provided *ad libitum* to a series of mice divided into four test groups (KD + Exercise, KD, Exercise, Control). In this study, anxiety and depression-like behaviors were evaluated using standard tests for measuring anxious behavior including the elevated plus maze (EPM), open field test (OFT) and forced swim test (FST). Levels of BHB were increased in all of the non-control test groups with no statistically significant differences between the three. Mixed results were seen in behavioral testing with the KD-Ex group overall yielding the greatest reduction in anxiety and depression-like behaviors. The elevated BHB of this group was also correlated with a lower LDL/HDL ratio, glucose, and insulin. Exercise, which has independently been shown to have positive effects on anxiety and depression in numerous preclinical and clinical studies, may be considered something of a confounding factor in this study, although efforts were made to address this by comparing a KD-exercise and a KD-sedentary group.

Similarly, an MCT diet was found to decrease anxiety-like behavior in rats (142). A diet of 5% MCT comprised of 40:60 octanoic acid triglyceride:decanoic triglyceride were fed to rats over a 15 day period. Rats were stratified based on anxiety-like behaviors as assessed via EPM testing and then submitted to social testing after being given an MCT diet. Rats on an MCT KD exhibited reduced anxiety behaviors in light–dark box testing and enhanced social competitiveness in social dominance testing. At the end of the diet, mitochondrial respiration was found to be reduced in the medial prefrontal cortex (mPFC) and unchanged in the nucleus accumbens of the test group. Enzymes associated with glycolysis and oxidative phosphorylation were also found to be decreased while proteins for glucose and glutamate transport were increased in the MCT diet group.

Gestational KDs also appear to have a neuroprotective effect on adult offspring. A study of 8-week old CD-1 mice exposed to the KD *in utero* and fed a non-KD postnatally were found to have a reduced susceptibility to anxiety-like behaviors as evaluated by open-field, forced-swim, and exercise wheel tests (143). Post-mortem MRI also found statistically significant neuroanatomical differences between the KD offspring and controls, with a 4.8% enlargement in cerebellar volume, a 4.8% reduction in corpus callosum volume, and a 1.4% hypothalamic reduction. The impacts of elevated KBs during development is an interesting prospect. It is known that compared to adults, for whom glucose is the dominant cerebral metabolic substrate, fetuses and newborns have proportionally lower cerebral glucose utilization and a greater capacity for utilizing alternative metabolic substitutes including lactic acid, BHB, AcAc, free fatty acids, and amino acids. KBs are typically lower in concentration in normal subjects and are thus contribute minimally to cerebral metabolism although utilization of lactate can exceed glucose usage in early postnatal life. The study provides some insight into the potential outcome of elevated circulating KBs via maternal KD feeding.

#### 4.2.2. Clinical

A single case study of the effect of a 68-year-old female with stage I Parkinson’s disease (PD) reported significant improvement in anxiety symptoms as assessed by the Parkinson’s Anxiety Scale (PAS) at 12- and 24-week follow-up but only minimal improvement in depression as assessed by the Centre for Epidemiologic Studies Depression Scale Revised-20 (CESD-R-20) (144). This was followed up by the same authors in a prospective study of 16 adults with PD who were fed a KD for 12-weeks (145). Supporting the results of the initial case study, statistically significant improvements were seen in anxiety scores as assessed by PAS but not in depression. PD-specific symptoms as assessed by the United Parkinson’s Disease Rating Scale (UPDRS): Parts I-IV were also evaluated with improvements in UPDRS: Part I scores, which assesses non-motor symptoms (i.e., mentation, behavior, and mood), a finding supported by a separate study evaluating the safety of the KD in PD patients (146).

Neuropsychiatric symptoms are feature of PD, becoming more florid and complex during the later stages of the disease. Anxiety and depression are common and not necessarily tied to disease progression, although it is generally thought that their pathophysiology is physiologically linked to PD rather than being simply a psychological reaction to the diagnosis. It is uncertain to what extent the pathophysiology of PD-associated anxiety differs from non-PD-associated anxiety. However, it is interesting to note that the KD appears to reduce anxiety symptoms in a complementary manner to preclinical studies of ketone supplementation in non-PD study groups, indicating that metabolic dysfunction might indeed be a universal common denominator to many of these conditions.

### 4.3. Evidence of exogenous ketones for treatment

Kovacs et al. have demonstrated the efficacy of exogenous ketone supplementation in alleviating anxiety-like behavior in rats since 2016 (147–150). In their 2016 study, ketones (including a low-dose ketone ester, high-dose ketone ester, BHB salt, and MCT) were orally supplemented both chronically in chow for one test group mimicking a KD and via oral gavage for another group (148). Confirmed elevation of BHB levels were recorded in all test groups compared to the control. EPM was used to evaluate anxiety-like behaviors. Based on behaviors in the arms of the maze (e.g., time spent in open arms, distance travelled in open arms, delayed latency to entrance of arms), ketone supplementation was associated with a reduction in anxiety-like symptoms in both chronic and sub-chronic feeding regimes. This was confirmed in a similar study that combined the use of video tracking software and yielded similar results (147).

In an extension of this work, the role of the adenosinergic system in the pathomechanism of anxiety was explored by attenuating adenosine A1 receptors directly (149). An A1R antagonist DPCPX was administered following intragastric gavage administration of a ketone salt-MCT supplement. It was found that while BHB levels remained high following administration of the ketone supplement, the improved performance in EPM demonstrating a reduction in anxiety-like behaviors was found to decrease significantly, with an apparent dose-dependent effect. This implies that A1Rs and the broader adenosinergic system may be responsible for modulating the overall anxiolytic effect of ketone supplementation.

Subcutaneous injection of BHB also yielded an improvement in anxiety-like behaviors in post-traumatic stress disorder (PTSD) rat models during EPM testing (151). Placing greater focus on the potential role of inflammasomes, Yamanashi et al. (151) explored the impact of BHB on TNF-α and IL-1β and found that BHB was able to partially attenuate increases of TNF-α induced by single prolonged stress (SPS). This lends greater weight to the idea that BHB appears to act as an inhibitor of inflammatory cytokines.

## 5. Schizophrenia

### 5.1. Disease overview

Schizophrenia is a serious, long-term mental syndrome primarily defined by episodes of psychosis. Global incidence is thought to range from 0.3 to 1% of the population with notable studies demonstrating substantial variability in its incidence across different population groups (152–154). It is associated with particularly poor outcomes including higher suicide rates (155), excess early mortality (156), lower quality of life (157), and a high economic burden through unemployment and loss of productivity (158).

Symptoms of schizophrenia are commonly grouped into positive and negative symptoms, with the former referring to changes in behavior and thought patterns and the latter referring to the diminishment of normal behaviors pertaining to socialization, motivation, and emotional expression. The most common, but not only, presentation of positive symptoms in schizophrenia are auditory hallucinations, paranoid or persecutory delusions, and disorganized speech. Negative symptoms occur in around 60% of patients and are often responsible for the poor long-term functional outcomes associated with schizophrenia (159). A third group of symptoms sometimes specified are cognitive symptoms, which refer to cognitive deficits in neuro and social cognition.

A landmark discovery made serendipitously in the 1950s are first-generation or typical antipsychotics, including chlorpromazine, haloperidol, and loxapine, which work as dopamine D_2_ receptor agonists to produce a tranquilising effect. While they remain a key treatment strategy today, they are notorious for inducing adverse extrapyramidal symptoms (EPS), which include akathisia, dystonia, and parkinsonism. For this reason, they have been somewhat superseded by second-generation atypical antipsychotics, including clozapine, olanzapine, quetiapine, risperidone, and aripiprazole, which act more broadly on dopamine (D_2_), serotonin (mainly 5-HT_2A_), and norepinephrine (α, β) receptors to produce a therapeutic effect. Antipsychotics appear to have variable success in treating schizophrenia’s broad profile of symptoms. Positive psychotic symptoms are often well treated by antipsychotics, as are secondary EPS or depressive symptoms, which can be managed by tailored management strategies extending beyond antipsychotics (e.g., SSRIs). However, primary negative symptoms do not always appear to respond well to current D_2_ antagonist antipsychotic treatments as reflected by the relatively high 30–40% of patients who do not respond adequately to treatment (160). This means that beyond the resolution of acute or episodic psychotic symptoms, it is common for patients with schizophrenia to face ongoing struggles with maintaining normal function and integrate successfully with society. As such, there is a need for alternative treatment strategies that can better address the full gamut of symptoms associated with schizophrenia.

While its pathophysiology is still not fully understood, a series of hypotheses seeking to explain the constellation of symptoms associated with schizophrenia have been posited over the years. The most well-known emerging during the latter half of the 20^th^ century, is the dopaminergic hypothesis, which focuses on abnormal dopamine expression, largely in response to studies of the psychosis-inducing effects of drugs like amphetamines as well as the development of dopamine attenuating drugs like chlorpromazine (161), which appeared to treat positive symptoms of the disease. While early results supported the notion that excessive dopamine played a central role in schizophrenia, later studies produced contradictory results. Clozapine, for example, one of the most effective drugs for treatment resistant patients was shown to have a relatively low affinity for D_2_ receptors, and a study of cerebrospinal fluid in schizophrenic patients revealed normal levels of dopamine metabolites (162). This ongoing work has led to a more finessed perspective on the role of dopamine, which now views dopaminergic dysfunction as a common denominator in a series of other contributing mechanisms. Other key hypotheses are the glutamatergic hypothesis, which focuses on disturbances in glutamatergic neurotransmission especially via NMDA receptor function, the serotoninergic hypothesis, the GABAergic hypothesis, and the contribution of inflammatory and oxidative stress (163, 164).

A recent review by Henkel et al. (165) draws attention to an alternative perspective of schizophrenia as a disorder of dysfunctional cerebral bioenergetics (165). This hypothesis builds on recent studies providing consistent and identifiable trends in metabolic dysfunction in schizophrenia including evidence of an increased risk of insulin resistance (166), impaired insulin signaling and glucose metabolism (167–173), dysfunctional astrocyte-neuron coupling leading to impaired lactate shuttling and glycolysis (174–179), and abnormal expression of creatine kinase (180, 181). This has led to identification of fundamental disruption in key metabolic cycles including the pentose-phosphate pathway (PPP), the tricarboxylic acid cycle (TCA), and oxidative phosphorylation (165). In some cases, GWAS studies have been able to identify higher risk polymorphic genes for these metabolic cycles (182–184). Further, there is a growing body of work demonstrating the effects of antipsychotics on cerebral metabolism both globally and in specific regions of the brain thanks to imaging studies (185–191).

This growing understanding of the metabolic dysfunction seen in schizophrenia lends greater weight to historical experimental studies demonstrating the efficacy of non-pharmacological treatments such as the KD (192) or regulation of gut-brain axis with probiotic supplementation (44). In particular, the role of KBs and their derivatives (i.e., acetyl-CoA) are of interest for their role as ATP precursors in the TCA cycle as well as the inflammasome inhibiting properties of BHB. More globally, it has been theorized that ketosis might be able to decrease glutamate:GABA ratios in the brain, attenuating GABA activity and thus altering neuronal activity. The following section reviews preclinical and clinical studies relating to the role of KBs and KDs in the pathophysiology and treatment of schizophrenia.

### 5.2. Altered ketone metabolites in schizophrenia

Metabolomics studies have shown that the overall metabolic profiles of schizophrenia patients are demonstrably altered and differentiable from controls (193). Early proof-of-concept studies have demonstrated that schizophrenic patients can be accurately identified based on profiles of as few as four metabolomic biomarkers alone (194–196), which represents a compelling new perspective on diagnosis possibilities. Most recently, 86 differential metabolites and over 180 differential expression genes have been identified in a recent transcriptomic analysis study (197), with pathway-based integration indicating that the synthesis and degradation of KBs appears to be closely connected to schizophrenia pathogenesis.

BHB has been found to have altered expression in schizophrenia patients. A 12-week clinical study of 38 age- and gender-matched patients found that baseline levels of BHB were higher in schizophrenia patients than in controls both at the beginning and end of the follow-up period (198). Further, this baseline was also found to exhibit a decreasing trend in patients undergoing aripiprazole or ziprasidone treatment. Significantly elevated BHBs were also observed in an earlier cross-sectional case–control study of 54 age- and gender-matched schizophrenic patients (199), which also recorded elevated pyruvate in patients treated with olanzapine and clozapine compared to other antipsychotics. In both instances, elevated BHB indicates impaired energy utilization in schizophrenia and could potentially be used to evaluate disease severity if supported by larger and longer longitudinal studies. These studies present an interesting avenue of research with BHB potentially being used as a biomarker for disease status.

The intriguing role of BHB has been elucidated in a particularly interesting study by Youm et al. (20), which found that BHB but not AcAc inhibits NLRP3-mediated inflammatory disease. NLRP3 is an inflammasome that triggers IL-1, a cytokine that is implicated in the cytokine hypothesis of schizophrenia pathogenesis (200). Increased or abnormal NLRP3 and IL-1 expression along with other specific cytokines including epidermal growth factor (EGF), IL-6, and neuregulin-1 have widely been positively associated with more extreme schizophrenia-like behavior (201). Notably, studies aiming to therapeutically target NLRP3 have found that BHB appears to indirectly inhibit NLRP3 (20), which led to the development of a MCC950/CRID3 (202), a NLRP3-specific inhibitor. Specifically, a BHB concentration of 5–10 mM has been reported as being sufficient to inhibit NLRP3 in murine macrophages, which is comparable to circulating concentrations observed in subjects complying with a KD. Of its diverse metabolic functionality, it appears that BHB’s ability to prevent K^+^ efflux in response to NLRP3 activators, suppress ASC speck formation, and limit IL-1β secretion in cells with gain-of-function NLRP3 mutations are of greatest interest in understanding its ability to inhibit NLPR3. It is still uncertain whether K^+^ channels are directly or indirectly inhibited by BHB. However, the potential therapeutic effect of BHB in this capacity of interest as it is comparably inexpensive as a therapeutic option when compared to the cost of producing IL-1 blocking antibodies or receptor antagonists.

### 5.3. Evidence of the KD for treatment

#### 5.3.1. Preclinical studies

Schizophrenia can be pharmacologically modelled in rodents by administering the NMDA-antagonist MK-801 or dizocilpine to induce schizophrenia-like behavior via NMDA receptor hypofunction in the prefrontal cortex (203). The first study utilizing the NMDA hypofunction model of schizophrenia in mice by Kraeuter et al. found that the KD in mice could attenuate pathological behaviors induced by MK-801 (204). They reported significant improvements in hyperactivity, stereotyped behaviors, ataxia, social withdrawal and spatial working memory impairment, which represent the positive, negative, and cognitive symptoms of schizophrenia. KD was also found to prevent impaired prepulse inhibition (PPI) of startle in sensorimotor gating tests (205). PPI of the startle refers to the tendency of a lower intensity stimulus to reduce the startle reflex triggered by a higher intensity stimulus, for example if a startling sound is followed immediately by a subthreshold acoustic stimulus. Impaired PPI has been shown to be an endophenotype in schizophrenic patients (206) and can be used as a measure of sensorimotor gating. When feeding male rats with a KD over a 7 week period and confirming ketosis with serum BHB levels, the study found that MK-801-induced PPI impairments were prevented at both 3 and 7 weeks. This was found to be the case irrespective of whether the test group was in a caloric deficit, which was evaluated in response to previous studies that have demonstrated that fasting can have benefits on neuroplasticity and brain health (207). The work supports the notion that schizophrenia’s pathophysiology may be responsive to metabolic-focused therapies.

Kraeuter et al. also evaluated the effects of the KD in conjunction with olanzapine treatment focusing on the same impaired PPI of startle during sensorimotor gating tests (208). A test group being treated with olanzapine alone, a group being treated with the KD alone, and a group being co-administered olanzapine and the KD were interrogated during sensorimotor testing. The study found that the KD was as effective as olanzapine in reducing MK-801-induced PPI impairments after 8 weeks of treatment. The study also found that the combined therapy was as effective, but not more, as the treatments administered individually, indicating that the two options could potentially be used together without causing deleterious effects on treatment.

#### 5.3.2. Clinical Studies

The KD has historically been noted to reduce schizophrenia symptoms as reported in clinical studies noting cravings for carbohydrates in patients shortly before episodic exacerbation of the illness (209). There is also interesting historical evidence for decreased admissions for schizophreniform illnesses in particular populations that may incidentally have been utilizing KDs, including European countries post-WW2 with reduced supplies of cereal-based foods (210) and South Pacific nations with a traditionally low dietary intake of grains (211). Connections between schizophrenia and the seemingly disparate condition coeliac disease have also previously been reported with a drastic reduction of schizophrenic symptoms being observed following gluten withdrawal (212). Dietary insufficiencies in the form of niacin, vitamin C, and folate deficiencies may also potentially exacerbate schizophrenic symptoms (213), suggesting a complex bioenergetic aspect to its pathophysiology.

Case reports of KD abruptly resolving instances of longstanding schizophrenic symptoms provide significant hope for treatment-resistant patients (214–217). Kraft and Westman (215) detailed the case of a 70-year-old woman who experienced remission of audiovisual hallucinations that had previously caused at least five episodes of hospitalization due to suicidality over the preceding 6 years despite trying lithium, olanzapine, ziprasidone, aripiprazole, quetiapine, haloperidol, lamotrigine, perphenazine, and risperidone prior to 2008. Palmer et al. (217) later described the resolution of a further episode of paranoia, disorganized speech, and audiovisual hallucinations in the same patient (then 82-years-old) within 2-weeks of starting a KD. Another case of 39-year-old woman with similarly treatment-resistant schizophrenia whose symptoms remitted after 1 month complying with a KD, enabling cessation of over 10 different medications she had been taking. In an earlier case report, Palmer (216) also reported on a 33-year-old man who experienced a dramatic reduction in auditory hallucinations and delusions after following a KD and also presented with dramatically worse positive and negative symptoms 1–2 days on five separate occasions after lapsing from the KD, as well as a 31-year-old woman who showed an marked improvement in symptoms within a month of starting the KD and similar concomitant worsening of persecutory delusions and paranoia during an episode where she broke the KD.

In all case studies, patients who had long histories of severe schizophrenia necessitating ongoing juggling of different drug permutations saw a decrease in positive and negative symptoms as assessed by PANSS after starting a KD, with the effects seemingly correlating with a current state of ketosis. As the patients all followed their own variations of the KD, further large scale research is required to determine associations between factors such as gluten intake, optimal macronutrient ratios, long-term efficacy, and tolerability of the diet. However, they provide a compelling clinical basis of the potential therapeutic value of the KD, particularly in light of the severity of symptoms that appear to have been ameliorated.

#### 5.3.3. Using the KD to prevent antipsychotic-induced hyperglycaemia

A common side-effect in patients on antipsychotic therapies is antipsychotic-induced hyperglycaemia, where the patient’s plasma glucose becomes elevated. Prolonged states of hyperglycaemia are undesirable as they can lead to the development of new-onset diabetes mellitus (DM) and the potentially life-threatening condition diabetic ketoacidosis. A relationship between atypical antipsychotics and both hyperglycaemia and DM is widely acknowledged (197, 218), with proposed mechanisms for its pathogenesis including insulin resistance, decreased insulin secretion from β-cells, and dysfunctional leptin action. Considering atypicals are used widely in the treatment of not only schizophrenia but also first-episode psychosis (FEP), severe depression and anxiety, and the management of manic episodes in bipolar disorder, this can present a challenging issue in clinical practice. Preclinical studies have demonstrated insulin resistance in acute usage of quetiapine, which can be mitigated via dietary supplementation of cholecalciferol or vitamin D3, implying modulated PI3K function (219).

A recent preclinical study found that the KD or short-term fasting can be used to protect against acute hyperglycaemia secondary to olanzapine usage independent of whole-body insulin usage (220), with blood glucose increasing in the chow-fed group but not the KD-fed group. Olanzapine was also found to increase serum glucagon in chow-fed mice more than in KD-fed mice, as well as increase phosphorylation of liver PKA substrates in chow-fed mice but not in KD-fed mice. Notably, the study found that exogenous supplementation of either oral BHB or ketone esters did not elicit the same therapeutic effect. The authors speculated whether the KD may have resulted in an overall higher concentration of circulating KBs and that exogenous supplementation may not have been enough to induce protective effects.

### 5.4. Evidence of exogenous ketones for treatment

Having previously demonstrated that the KD could reduce PPI of startle in MK-801-induced schizophrenia models, Kraeuter et al. extended this work by evaluating the effects of exogenous ketone treatment (221). Following administration of MK-801, animals were subjected to either acute intraperitoneal injection of BHB (2, 10, or 20 mmol/kg) or a 3-week chronic lower dose injection (2 mmol/kg) of BHB before being evaluated with tests. In addition to evaluating PPI of startle, the 2020 study also used open field testing to evaluate exploratory locomotor activity and social interaction including anogenital and intrafacial sniffing, pursuit, and active avoidance. The expanded scope of testing was intended to model both positive and negative symptoms of schizophrenia. BHB levels were significantly elevated in both the acute and chronic dose groups. Locomotor hyperactivity, deficits in social behavior, and impaired PPI of startle, all baseline MK-801-induced behaviors representing a schizophrenia-like tendency, were normalized or reduced in both the acute (10 and 20 mmol/kg dose) and chronic BHB treatment groups. The study acts as a proof-of-concept in demonstrating that exogenous KB treatment could be a feasible therapeutic option and yields comparable results to the KD.

## 6. Conclusion

Over the last century there has been fluctuant interest in ketogenic process involvement in the pathophysiology and treatment of psychiatric disorders. The enhanced neuroscientific understanding of neuropsychiatric disorders gleaned in the most recent decades as well as an improved insight into the biochemical interactions of KBs lend a stronger backbone to existing qualitative observations regarding the apparently neuroprotective effect of the KD. The following review has discussed usage of the KD and exogenous ketones in the treatment and understanding three major psychiatric conditions. A compelling body of preclinical evidence in support of KBs involvement and their potential for future therapeutics exists. Certainly, preclinical studies of psychiatric disease are intrinsically marred by the inability to replicate psychosocial contributors. It can also be argued that many of the tests used such as immobility testing (e.g., TST, FST) do not reflect depressive behavior and are often adaptive (222), becoming rather a reflection of active versus passive behavior in response to acute stress. For example, tail climbing behavior in certain strains of mice like C57BL/6 effectively renders the TST useless (105). Despite this, the promising results coupled with the relative simplicity of either the KD or exogenous ketone supplementation as a treatment option means that further clinical work is warranted.

At present, clinical studies are scarce with systematic controlled trials in this topic area being almost non-existent. This lack of well-designed and well-powered human studies leaves the therapeutic effectiveness of KBs still poorly understood. Particular research questions warranting targeted investigation include: (1) a direct comparison of the therapeutic effectiveness of KDs versus exogenous ketone supplements; (2) a comparison of KB effectiveness in psychiatric patients with and without known comorbid metabolic disorders (e.g., diabetes); and (3) broader characterization of neurobiological changes in subjects exhibiting a positive response to ketone therapy. It is curious why ketone therapy in neuropsychiatry remains so under-researched, especially in light of the array of commercially available ingestible ketone preparations available that have all been shown to have excellent tolerability, safety, and efficacy in inducing nutritional ketosis. Various answers to this question could be proffered including early decisions to position commercial ketone formulations as performance enhancing drinks for mass consumption, the traditional notion of the mind–body duality that continues to pervade the clinical subconscious and plague psychiatry as a discipline, the typically challenging nature of ethical approval in psychiatric research, and a perception from pharmaceutical companies that novel drug development for psychiatry is not as profitable as other specialties.

Despite this, the current authors argue in favor of further experimental work into this topic. Studies elucidating the metabolic perspective of psychiatric disease are well-aligned with the direction of modern psychiatry, which is increasingly migrating towards a more integrated model of psychiatric disease, lending equal weight to both biological (e.g., genetic, developmental, and inflammatory) elements and traditionally recognized psychosocial elements. Current pharmacological-based standard of care have limitations and often carry risky side-effect profiles. Thus, researchers in this area can benefit from the existence of ketone supplements that already comply with FDA guidelines and are classified as generally regarded as safe (GRAS). The primary aim of early clinical studies would be to establish whether the therapeutic effects observed in preclinical work are also observable in patients, with further work subsequently addressing factors like optimal dosing and drug interactions.

## Author contributions

NO wrote and compiled the draft. MM and GW provided text edits on clinical and metabolic aspects, respectively. LM provided text edits and oversight of the review direction. All authors contributed to the article and approved the submitted version.

## Conflict of interest

LM is the Research Lead of Health via Modern Nutrition Inc. (H.V.M.N.), which develops and commercializes products based on ketosis. GW is the founder and Executive Chairman of H.V.M.N. NO is a scientific writer for H.V.M.N.

The remaining author declares that the research was conducted in the absence of any commercial or financial relationships that could be construed as a potential conflict of interest.

## Publisher’s note

All claims expressed in this article are solely those of the authors and do not necessarily represent those of their affiliated organizations, or those of the publisher, the editors and the reviewers. Any product that may be evaluated in this article, or claim that may be made by its manufacturer, is not guaranteed or endorsed by the publisher.
